# Reply to “Comment on ‘De Novo Reconstruction of 3D Human Facial Images From DNA Sequence’”

**DOI:** 10.1002/advs.73979

**Published:** 2026-01-28

**Authors:** Mingqi Jiao, Jiarui Li, Chunman Zuo, Sijia Wang, Luonan Chen

**Affiliations:** ^1^ Key Laboratory of Systems Health Science of Zhejiang Province Hangzhou Institute for Advanced Study Chinese Academy of Sciences University of Chinese Academy of Sciences Hangzhou China; ^2^ CAS Key Laboratory of Computational Biology Shanghai Institute of Nutrition and Health Chinese Academy of Sciences University of Chinese Academy of Sciences Shanghai China; ^3^ School of Life Sciences SunYat‐sen University Guangzhou China; ^4^ School of Mathematical Sciences and School of AI Shanghai Jiao Tong University Shanghai China

## Abstract

This manuscript is a formal response to the Comment by Wagner et al. regarding our publication “De Novo Reconstruction of 3D Human Facial Images from DNA Sequence.” We clarify the methodological rationale, analytical procedures, and scientific scope of our original study, and we address several misconceptions arising in the Comment. We further highlight the intended purpose and limitations of Difface and discuss ongoing efforts to advance the rigor, interpretability, and ethical governance of DNA‐based 3D facial prediction.

We sincerely thank the authors of the Comment for their interest in our work and for initiating a scientific discussion on DNA‐based facial reconstruction [1, 2]. The Comment raises several points related to benchmarking practices, reconstruction error interpretation, demographic baselines, SNP deletion experiments, and the independence of training and testing sets. These topics are important for the development of rigorous standards in this emerging field, and we welcome the opportunity to clarify our methodology and correct inaccuracies.

## 1 Clarification on the Evaluation Framework and Methodological Approach

The Comment suggests that a linear PLSR (Partial Least Squares Regression) trained on European faces achieves lower error and therefore questions the magnitude of our demographic‐only evaluation. Actually, linear baselines such as PLSR face a significant limitation: they tend to regress toward an average face, producing deceptively low reconstruction errors while failing to capture individual‐specific characteristics. This underscores the limitation of relying solely on a single average reconstruction error as the main metric. For example, in facial feature classification tasks, reconstructions from linear methods show markedly lower accuracy compared to individual‐specific ground‐truth faces. In contrast, the original Difface study explicitly addressed these concerns by employing multiple complementary evaluations, including per‐vertex reconstruction errors, classification tasks comparing generated and real faces, direct visual assessments, diversity analysis, and robustness tests under different SNP input densities, was employed in our paper (see Figure 3.d for per‐vertex errors, Figure 4.a for classification tasks, Table 2 for direct visual assessments, Table 3 for diversity analysis and Figure 5 for robustness tests). These complementary metrics could provide a more complete picture of generative performance under varied conditions. The central objective in facial reconstruction is the accurate preservation of individual identity and key facial features (which are considered a small part of all facial features among all 7,906 spatially dense landmarks), as these are most strongly tied to genetic information and relevant to downstream applications such as recognition or forensics. Average reconstruction error, which is largely driven by global face shape or all facial features rather than those key facial features, cannot individually capture identity‐specific or genetically driven variation. A robust assessment therefore requires multiple complementary metrics.

Moreover, cross‐population comparison (European vs. Han Chinese) is scientifically inappropriate due to differences in facial shape variance, LD (Linkage Disequilibrium) structure, and covariate distributions. These factors explain why raw error values cannot be compared across modeling paradigms or populations.

## 2 Clarification on Dataset Partitioning and Addressing Concerns Regarding Potential Data Leakage

We appreciate the concern raised regarding the potential violation of data independence in our study. However, this interpretation does not accurately reflect the design of our study. The 7,842 SNPs used in our model were derived from prior Genome‐Wide Association Study (GWAS) results, which had undergone rigorous discovery and replication across independent cohorts [3]. Specifically, these SNPs were obtained not from all three cohorts (totaling 9,674 samples), but from two independent cohorts (6,968 samples for discovery) and were validated in a separate cohort (2,706 samples).

In this design, the SNPs were treated as fixed prior features. The model was then built using a two‐stage training/validation approach: first, SNPs were selected based on the training and testing cohorts (stage 1), and then the models were built by randomizing the training and testing samples in the second stage. Importantly, the test set was exclusively reserved for evaluation and validation, and no phenotypic data from the test set were used during model training. This two‐stage process, which involves using prior validated SNPs, is a reasonable and widely accepted strategy for small‐sample datasets like ours (9,674 total samples).

## 3 Clarification on the Interpretation of SNP Deletion and Robustness Evaluation

The SNP deletion experiment warrants another clarification. Our objective was not to infer genetic causality, but to evaluate the robustness of a machine‐learning model under missing genomic inputs, a realistic scenario for forensic and clinical applications. Random SNP deletion is a standard robustness evaluation analogous to dropout or feature masking. While locus‐level deletion may provide additional biological insights in future studies, the absence of such an analysis does not invalidate the conclusions drawn from our robustness assessment.

Several factual points also merit correction. The proposed “random–average–nearest neighbor” framework is novel and not a community standard, so applying it retroactively to Difface may lead to misinterpretation, particularly due to differences in population structure and modeling objectives. While we agree that millimeter‐level error alone cannot determine identity recognizability, our article highlighted Difface's methodological limitations and emphasized the need for caution in any forensic or clinical applications. Nonetheless, reconstruction error remains a valid measure of generative fidelity within the appropriate context. Additionally, our Methods clearly describe that 3D facial meshes were standardized to 7,906 dense landmarks via MeshMonk [4] registration, followed by rigid Procrustes alignment that preserves physical scale, making our millimeter‐scale reconstruction error accurate.

As detailed in our publication, we emphasized that Difface remains a scientific tool rather than a system suitable for operational or case‐level use. We also explicitly addressed the ethical, social, and privacy considerations associated with DNA‐based face modeling, underscoring the need for great caution, transparent communication of limitations, and appropriate regulatory safeguards before any real‐world application can be contemplated.

## 4 Data and Code Availability, Ethical Considerations, and Clarification of Data Access

We would like to clarify that the meta‐analysis GWAS summary statistics from this study are publicly available through the National Omics Data Encyclopedia, i.e., NODE:OEP002283 (https://www.biosino.org/node/project/detail/OEP002283). In addition, the code for the model used in this study has been publicly released on GitHub: https://github.com/Jiao‐mq/Difface. For further technical details can be requested from M.J.

The participants making up the NSPT, NHC and TZL datasets were not collected with broad data sharing consent. Given the highly identifiable nature of both facial and genomic information and unresolved issues regarding risk to participants, we opted for a more conservative approach to participant recruitment. Broad data sharing of the raw data from these collections would thus be in legal and ethical violation of the informed consent obtained from the participants. This restriction is not because of any personal or commercial interests. Additional details can be requested from L.J. for the NSPT dataset, and S. Wang for the NHC and TZL datasets. Data usage shall be in full compliance with the Regulations on Management of Human Genetic Resources in China. We recognize the importance of open science and regret that full data sharing is not possible in this instance.

In summary, what began as an exploration of nonlinear genotype–phenotype mapping resulted in the development of Difface—a diffusion‐based framework enabling facial reconstruction from genetic variants. We are grateful for the opportunity to clarify our study, address the points raised in the Comment, and contribute to the continued progress of genetically informed facial modeling and its responsible translation.

## Conflicts of Interest

The authors declare no conflicts of interest.
